# Stroke Severity Versus Dysphagia Screen as Driver for Post-stroke Pneumonia

**DOI:** 10.3389/fneur.2019.00016

**Published:** 2019-01-29

**Authors:** Thanh G. Phan, Talvika Kooblal, Chelsea Matley, Shaloo Singhal, Benjamin Clissold, John Ly, Amanda G. Thrift, Velandai Srikanth, Henry Ma

**Affiliations:** ^1^Stroke and Ageing Research (STARC), Department of Medicine, School of Clinical Sciences at Monash Health, Monash University, Clayton, VIC, Australia; ^2^Department of Neurology, Monash Health, Monash University, Melbourne, VIC, Australia; ^3^Department of Medicine, Peninsula Health and Central Clinical School, Monash University, Melbourne, VIC, Australia

**Keywords:** stroke, pneumonia, NIHSS, dysphagia, decision tree

## Abstract

**Background and Purpose:** Post-stroke pneumonia is a feared complication of stroke as it is associated with greater mortality and disability than in those without pneumonia. Patients are often kept “Nil By Mouth” (NBM) after stroke until after receiving a screen for dysphagia and declared safe to resume oral intake. We aimed to assess the proportional contribution of stroke severity and dysphagia screen to pneumonia by borrowing idea from coalition game theory on fair distribution of marginal profit (Shapley value).

**Method:** Retrospective study of admissions to the stroke unit at Monash Medical Center in 2015. Seventy-five percent of data were partitioned into training set and the remainder (25%) into validation set. Variables associated with pneumonia (*p* < 0.1) were entered into Shapley value regression and conditional decision tree analysis.

**Results:** In 2015, there were 797 admissions and 617 patients with ischemic and hemorrhagic stroke (age 69.9 ± 16.2, male = 55.0%, National Institute of Health Stroke Scale/NIHSS 8.1 ± 7.9). The frequency of pneumonia was 6.6% (41/617). In univariable analyses NIHSS, time to dysphagia screen, Charlson comorbidity index (CCI), and age were significantly associated with pneumonia but not weekend admission. Shapley value regression showed that the largest contributor to the model was stroke severity (72.8%) followed by CCI (16.2%), dysphagia screen (3.8%), and age (7.2%). Decision tree analysis yielded an NIHSS threshold of 14 for classifying people with (27% of 75 patients) and without pneumonia (2.5% of 308 patients). The area under the ROC curve for training data was 0.83 (95% CI 0.75–0.91) with no detectable difference between the training and test data (*p* = 0.4). Results were similar when dysphagia was exchanged for the variable dysphagia screen.

**Conclusion:** Stroke severity status, and not dysphagia or dysphagia screening contributed to the decision tree model of post stroke pneumonia. We cannot exclude the chance that using dysphagia screen in this cohort had minimized the impact of dysphagia on development of pneumonia.

## Introduction

Post-stroke pneumonia is a feared complication of stroke as it is associated with greater mortality and disability than those without this complication. Post-stroke pneumonia occurs in ~10% of patients after stroke (1). Earlier small trials to prevent post-stroke pneumonia, including prophylactic administration of antibiotics, do not support a role for antibiotics in reducing mortality, or dependency (1). In a more recent and larger trial there was no evidence for an effect of antibiotics on reducing pneumonia (2). Further, patients receiving antibiotics had longer hospitalization than those in the usual care group. The use of nasogastric feeding to bypass swallowing difficulties does not appear to reduce the risk of pneumonia (3, 4).

The frequently used term aspiration pneumonia implies a direct link between dysphagia, aspiration, subsequent infection and pneumonia, but there are missing components in this pathway (5, 6). Swallowing difficulties on video-fluoroscopy have been described in substantial proportion (>39%) of elderly subjects without dysphagia (7). Dysphagia is common after stroke and occurs in ~37–93% of patients (8, 9). Screening for dysphagia has been emphasized as important for preventing pneumonia at both an international symposium (10) and in clinical audit reports (11). In the American Heart Association 2018 guidelines it was deemed to have strength/class of recommendation IIa (moderate benefit to risk ratio) but level of evidence C-LD (limited data) (12). In the Australian 2017 guidelines, the recommendation to perform a dysphagia screen after stroke was classed as consensus-based (13).

Patients are often kept “Nil By Mouth” (NBM) after stroke until a dysphagia screen has been performed and the patients is declared safe to resume oral intake. In our center, the decision to undergo dysphagia screen is *ad-hoc* and depends on the treating team. However, there are uncertainties about the utility of performing a screen for dysphagia (14–16). Most tools have high sensitivity but much lower specificity to detect swallowing difficulties (15). Because the tool is highly sensitive patients may be prevented from oral intake, including taking important medications such as antithrombotic therapy and antihypertensive therapy. The effectiveness of screening for dysphagia in preventing post-stroke pneumonia has been questioned in systematic reviews since 2010 (14–16). The aim of this study is to identify variables associated with post-stroke pneumonia, their proportional contribution and the thresholds for these associations.

## Methods

This is a retrospective study of admissions to the stroke unit at Monash Health over 12 months in 2015. We collected data on demographic variables, admission diagnoses, time to triage, imaging, screening for dysphagia, NBM status, stroke severity (National Institute of Health Stroke Scale/NIHSS), and Charlson comorbidity index (CCI). The NIHSS has values ranging from 0 (no signs) to 42 (intubated patient). In this study, the NIHSS was taken from the clinical examination performed on admission. In this study, CCI was calculated from the extracted clinical data (17). This study was approved by Monash Health Human Research Ethics Committee. A waiver of individual consent was granted given that the study was retrospective in nature and there was no intervention component to this study.

Swallowing assessment: following assessment by the medical team patients are designated into two groups: (1) to be kept NBM) until a dysphagia screen or; (2) able to swallow and take medications. The dysphagia screen was conducted by nurses certified to perform this task. The dysphagia screen tool used was Acute Screening of Swallow in Stroke or TIA (ASSIST) tool (18). Patients who passed the screen were permitted to have oral intake, while patients failing the screen remained NBM until reviewed by a speech pathologist within 24 h of admission. The speech pathologist then either allowed the patients to have a modified diet, or required that they remain NBM.

Definition: The diagnoses of TIA, minor stroke and stroke were confirmed by a stroke neurologist. In this study, TIA was defined as transient neurological weakness lasting up to 24 h and resulted from ischemia of the brain or eye (19). Patients in the minor stroke pathway had NIHSS ≤ 4 (20). In this study, the term post-stroke pneumonia will be used instead of aspiration pneumonia. The diagnosis of post-stroke pneumonia was made by the treating team. It was defined clinically by the finding of fever (temperature ≥38°C), consolidation on chest radiograph and use of antibiotic medications.

We used *lubridate* package in R (version 3.4.2) to parse time for weekend vs. weekday analysis. Time to event was plotted using R packages *survival* and *survminer*. Seventy-five percent of data were partitioned into a training set and the remainder (25%) into a validation set. Variables found to be associated with pneumonia (*p* < 0.1) in the training set were entered into multivariable regression, hierarchical partition analysis, and decision tree analysis.

Given the interest in finding out the covariate making largest contribution to Pneumonia, we calculated the marginal contribution of each covariate to the model or Shapley value regression (21, 22). The marginal contribution is determined as the average of all permutations of the coalition of the covariates containing the covariate of interest minus the coalition without the covariate of interest. This analysis was performed using *iml* package in R (23).

Further, we used conditional decision tree analysis to develop models for classifying between pneumonia and no pneumonia. Decision tree was trained on the training data and validated with the testing dataset. The tree construction was performed using the *party* package in R (24). Classification of a binary response variable can be viewed as having a set of rules that are applied sequentially, with each rule partitioning an attribute (predictor variable) into a binary response. The area under ROC curves were compared using *pROC* package in R.

## Results

Over the full 2015 calendar year there were 797 admissions and 617 patients with ischemic and hemorrhagic stroke (age 69.9 ± 16.2, male = 55.0%, National Institute of Health Stroke Scale/NIHSS 8.1 ± 7.9). The frequency of pneumonia was 6.5% (45/797) among the entire cohort and 6.6% (41/617) among patients with diagnosis of stroke. See Table 1 for the patient characteristics in each group. In this study, 75.8% (468/617) of patients were kept NBM, 77.8% (480/617) of the patients were screened for dysphagia that was documented in their medical records and 69.4% (333/480) passed. Among the patient who did not have dysphagia screen, only two had pneumonia. There was no statistically significant difference between the training and testing groups with respect to NBM, dysphagia screen, passing dysphagia screen, and pneumonia (Table 1). There was no difference in stroke severity between the training group (7.4 ± 7.7) than in the testing group (7.7 ± 7.2, *p* = 0.7). Twenty eight of 29 patients (96.5%) with pneumonia in the training group were kept NBM from onset compared to 7 of 8 (87.5%) in the test group. Eleven of 29 patients (37.9%) with pneumonia in the training group passed dysphagia screen compared to 4 of 8 (50.0%) in the test group (*p* = 0.5).

**Table 1:** Characteristics of patients with TIA, stroke, and hemorrhagic stroke.

	**Admission (%)**	**Ischemic and hemorrhagic stroke diagnosis**	**Hemorrhagic stroke**	**Ischaemic stroke (excluding TIA and minor stroke)**	**TIA**	**Minor stroke pathway**	**Non-stroke**	**Training**	**Testing**	***P*-value between training and testing**
N	797	617	92	436	42	47	180	393	132	–
NBM	534 (76.7)	468 (75.9)	77 (83.7)	348 (79.8)	16 (38.1)	27 (57.4)	66 (36.9)	290 (73.8)	101 (76.5)	0.5
Dysphagia screen	544 (78.0)	480 (77.8)	72 (78.2)	360 (82.6)	16 (38.1)	32 (68.1)	64 (35.7)	306 (77.8)	102 (77.2)	0.9
Pass dysphagia screen	383/544 (70.4)	333 (69.4)	47 (65.3)	244 (67.8)	15/16 (93.8)	27/32 (84.4)	50/64 (78.1)	214 (69.9)	72 (70.5)	0.9
Pneumonia	45 (6.5)	41 (6.6)	5 (5.4)	35 (8.0)	0 (0)	1 (2.1)	3 (1.7)	28 (7.1)	8 (6.1)	0.6
Age	69.9 ± 16.2	71.7 ± 15.2	71.1 ± 14.1	71.9 ± 15.9	73.2 ± 11.5	69.3 ± 12.8	63.7 ± 17.9	71.0 ± 15.4	74.3 ± 15.1	0.03
NIHSS	6.7 ± 7.6	8.0 ± 7.9	5.6 ± 2.7	8.5 ± 7.8	1.3± 1.4	2.7 ± 1.8	NA	7.4 ± 7.7	7.7 ± 7.2	0.7
Charlson comorbidity index	5.0 ± 2.8	5.5 ± 2.6	11.6 ± 9.2	5.6 ± 2.7	5.2 ± 2.1	4.9 ± 2.3	3.5 ± 2.7	5.4 ± 2.6	5.7 ± 2.6	0.5

Variables associated with pneumonia (*p* < 0.10) in univariable analyses included stroke severity, NBM, time to dysphagia screen, CCI, age, and week day vs. weekend admission. Tissue plasminogen activator (tPA) usage was not associated with pneumonia; among 52 stroke patients in training set who received tPA, only three had pneumonia among 33 stroke patients in test set who received tPA, only one had pneumonia. The regression analyses were similar for analyses of the full sample (patients with TIA, ischemic, and hemorrhagic stroke) and for patients with only TIA or ischemic stroke. The findings were the same and hence are presented together.

**Figure 1:**
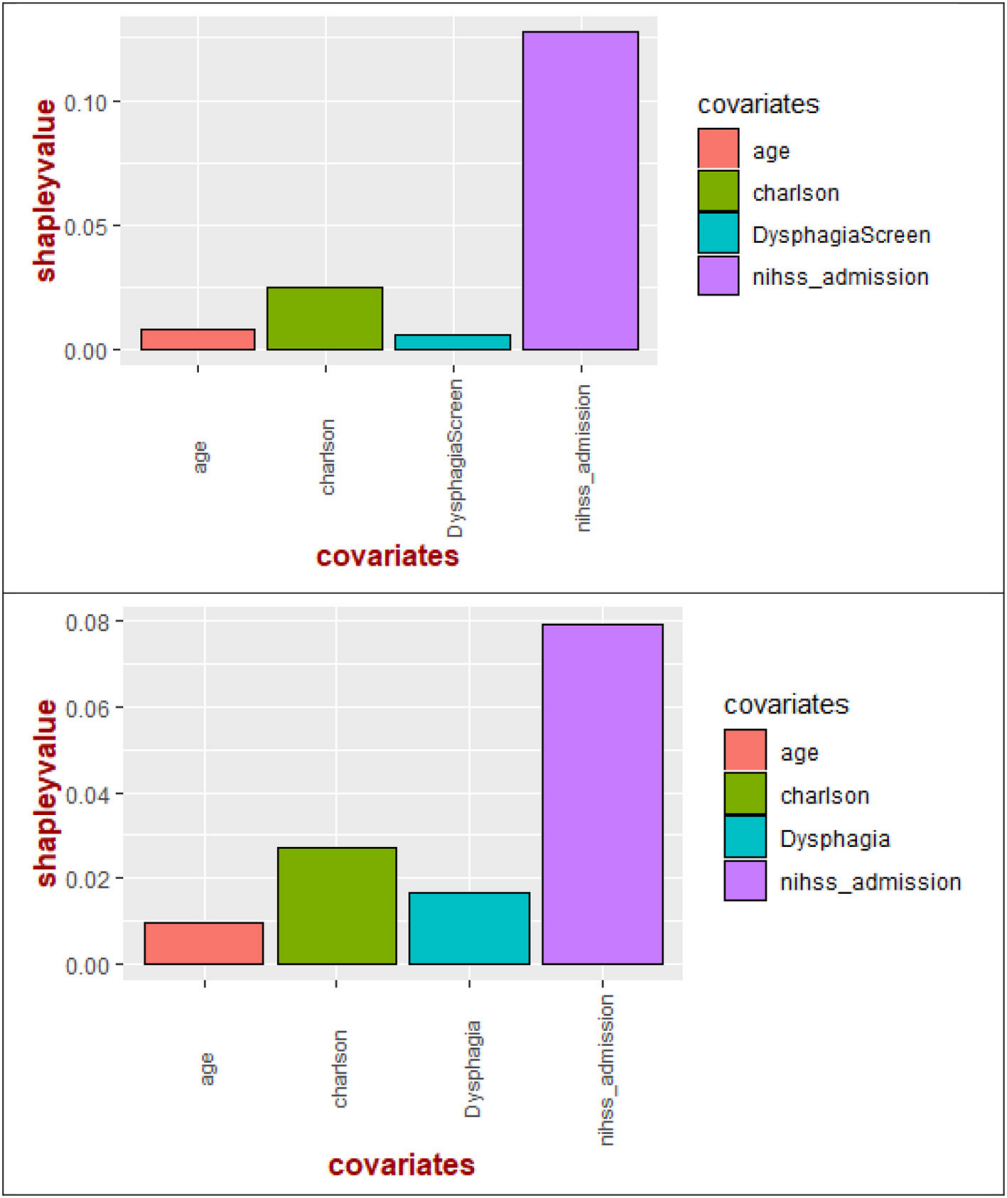
Shapley values of the covariates for pneumonia. Shapley value regression showed importance of stroke severity on admission above other covariates such as Charlson comorbidity index, age, dysphagia screen (or time to dysphagia screen). In the bottom figure, stroke severity has higher importance than Charlson comorbidity index, dysphagia, and age.

Significant variables (*p* < 0.10) described above were entered into multivariable regression and decision tree analysis. Shapley value regression showed that the largest contributor to the model was stroke severity (72.8%) followed by CCI (16.2%), dysphagia screen (3.8%), and age (7.2%) (Figure 1). The results remained the same when time to dysphagia screen was used instead of the term dysphagia screen or presence of dysphagia (see Figure 1).

**Figure 2:**
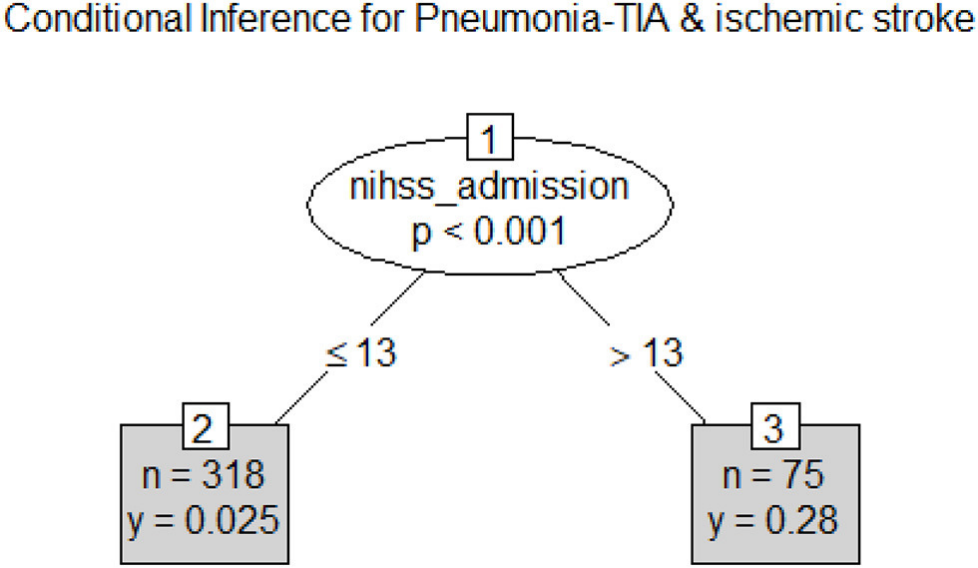
Classification tree for Pneumonia. Pneumonia can be classified on the basis of stroke severity with threshold at National Institute of Health Stroke Scale >13. The frequency of pneumonia was 0.28 among those with higher stroke severity and was 0.025 lower among those with lower stroke severity.

Decision tree analysis yielded an NIHSS threshold of 14 for classifying people with (27% of 75 patients) and without pneumonia (2.5% of 308 patients). The area under the ROC curve for training data was 0.83 (95% CI 0.75–0.91) with no detectable difference between the training and test data (*p* = 0.4); see Figure 2. Variables such as dysphagia, dysphagia screen, time to dysphagia screen, NBM, CCI, age, and weekday vs. weekend admission did not remain in the model.

**Figure 3:**
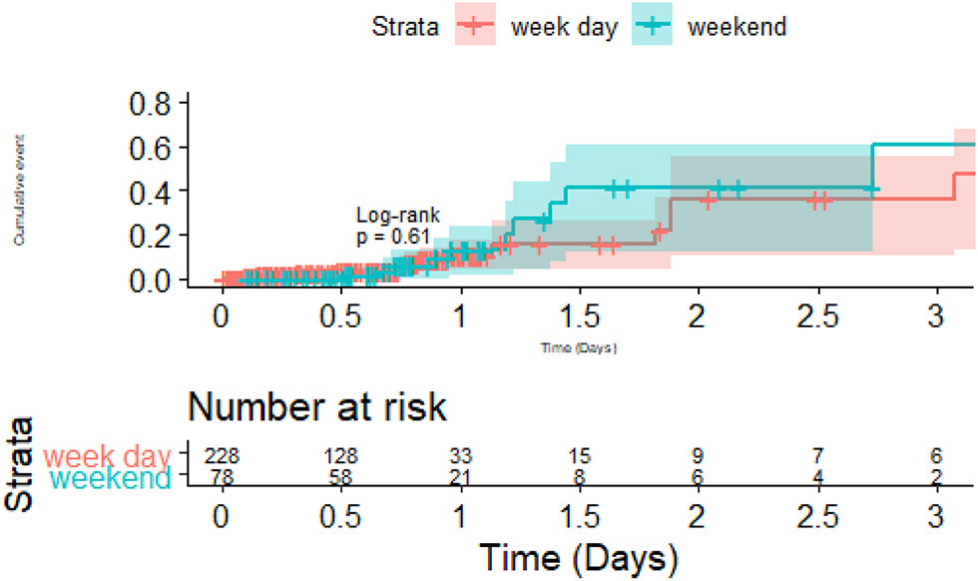
Dysphagia screen on weekend and week day vs. Pneumonia. There was no statistical difference in the pneumonia rate between weekday vs. weekend admission.

The survival plot was performed on the 393 patients with TIA and ischemic stroke in the training dataset; these patients were selected because they have documented time of dysphagia screen. In survival analysis of the 393 patients with TIA and ischemic stroke in the training dataset, the time to conducting a dysphagia screen was significantly different between the weekend and week day (*p* = 0.003; Figure 3). However, there was no difference in the frequency of pneumonia between weekend and week day (*p* = 0.4). Among patient who had dysphagia screen, 39.2% (120/306) of patients received a dysphagia screen within 12 h and 82.3% (252/306) within 24 h.

## Discussion

The key finding of this study was that stroke severity plays a critical role in post-stroke pneumonia. The Shapley value regression, based on ideas in co-operative game theory, showed that stroke severity had higher contribution to the variance of the data than that provided by dysphagia or dysphagia screen. Importantly, post-stroke pneumonia developed among patients who were kept NBM in our study. These findings emphasized that other factors not measured here contributed to the development of pneumonia. Factors not measured in this retrospective analysis and which could explain the findings are the relationship between stroke severity, immunosuppression, and pneumonia (25).

**Figure 4:**
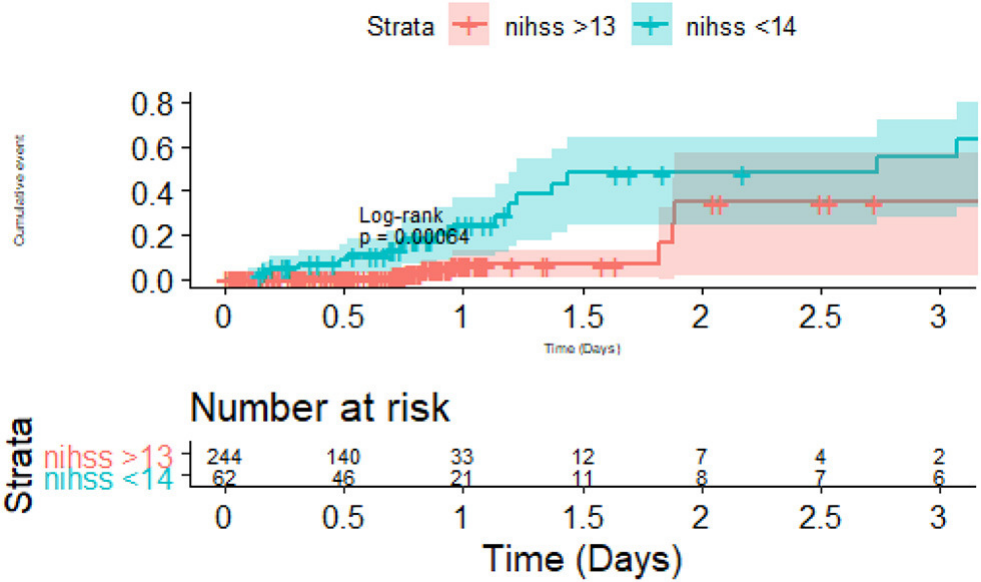
Importance of stroke severity and Pneumonia. There was difference in pneumonia between the group with severe stroke (National Institute of Health Stroke Scale >13) vs. the group with less severe stroke. The risk of pneumonia occurred in the first 2 days and then the risk of pneumonia plateaued between the two groups.

Post-stroke immune dysfunction has been associated with increasing volume of the infarct (from inflammatory cascade) and stroke severity (26). Immunosuppression, mediated in part by the sympathetic nervous system, may be the body's response to dampen the inflammatory response within the brain (25), but the unintended consequence is an increased risk of infection such as pneumonia (27). Recently researchers have described translocation and dissemination of commensal gut bacteria as novel pathway for post-stroke pneumonia (28). The bacterial dosage necessary to cause post-stroke pneumonia in animal with experimental stroke can be very small (29); this bacterial effect on animal model stroke was attenuated by the use of beta-blocker (block sympathetic nervous system). Consistent with this, researchers using the VISTA-Acute database have described a reduction in pneumonia with use of beta-blocker either before or after stroke onset (30). These findings may explain in part our findings that stroke severity (Figure 4) and not dysphagia screen was a major driver in post-stroke pneumonia in this cohort. Furthermore, these findings support those of a systematic review in which there was lack of impact by dysphagia screen in preventing post-stroke pneumonia (15, 16).

The role of dysphagia screen appeared to make intuitive sense, since avoiding oral food intake in patients with ineffective swallow theoretically prevent them from inadvertently inhaling food. Yet, performing dysphagia screen and keeping patients NBM on hospital admission in this study did not prevent them from having pneumonia. This point, that pneumonia occurred more often in patients kept NBM, is not often discussed in other studies (11, 31). Consistent with our findings, other investigators have described pneumonia occurring among patients with percutaneous endoscopic gastrostomy (6), nasogastric feeding (3), and contemplated the use of metoclopramide to prevent aspiration among these patients (32). These studies suggested that there are hidden factors at play in the development of pneumonia.

An important difference between our study and those of others is that our study was performed in one tertiary referral center. In contrast, other studies comprised audit data from large numbers of hospitals but have missing NIHSS in unavailable in a quarter (11) to half (33) of the patients. The authors consequently used level of consciousness (11) or other clinical variables (33) as a proxy for stroke severity in the regression analyses. The authors surmised that “having complete data on stroke severity may have strengthened the study” (11). Further, there was imbalance between the groups in terms of the stroke severity and making interpretation of the results difficult (33). It is also likely that there were differences in hospital practices which may account for differences in outcome but that this variable was not accounted for in the analysis. With a much larger database of patients, North American investigators from Get with the Guidelines found that the relationship between pneumonia and dysphagia screen was confounded by stroke severity as measured by NIHSS (31). This analysis was performed to assess the significance of the relationship between screening for dysphagia and pneumonia, but not to assess the strength of the relationship between dysphagia screen, NIHSS, and pneumonia; to do so would require a much different approach to traditional regression analysis. The different statistical approach is detailed below.

In this study we had used a variety of statistical tool to illustrate the key findings. In particular, we used ideas from game theory relating to fair distribution of profit in coalition games; the coalition (co-operative) game in this case can be interpreted as contribution of the covariates to the model (21). The Shapley value regression method calculates the marginal contribution of each covariate as the average of all permutations of the coalition of the covariates containing the covariate of interest minus the coalition without the covariate of interest. The advantage of this approach is that it can handle multicollinearity (relatedness) among the covariates (23); fortunately the regression diagnostics (variance inflation factor) did not show evidence of collinearity in this sample. A drawback of Shapley value regression is that there are no reports on odds ratio; rather the description is that of the proportional contribution of the covariates to the model (34). This method can be performed manually for small number of predictors with ease but can become computational difficult for very larger number of predictors due to the calculation of permutations of the covariates.

We supplement this analysis by performing conditional decision tree analysis. This method has similarity to clinical pathway and ease of understanding by clinicians (35). A typical decision tree resembles a logical flow chart diagram (2). The tree is grown using a “divide and conquer” strategy, with repeated partitioning of the original data into smaller groups (nodes) on a yes or no basis. Our approach is superior to CART method because that method uses information criterion for partitioning and which can lead to overfitting (36). The scenario of overfitting describes model which works well on training data but less so with new data. The conditional approach by *party* is less prone to overfitting as it includes significance testing (24). In this analysis, the area under to ROC curve for stroke severity as a tool for discriminating between pneumonia and no pneumonia can be classified as excellent (37). The fact that we found no significant difference in the results for those partitioned into training and testing groups supported the stability of our model. The critical threshold for pneumonia in this study was around NIHSS of 14.

## Limitations and Strengths

This is a retrospective study from a single institution consisting of patients over a 12-month period. One advantage of a single center approach is that we have a known path for management of swallowing compared to an audit where the type of swallowing assessment is not known (11). The frequency of pneumonia in our study was similar to that in other studies in North America (unscreened 4.2%, screen and passed 2%, screened and failed 6.8%) (33), England (8.7%) (11), and Virtual International Stroke Trials Archive/VISTA cohort (8.2%) (30). While the sample here comprises several hundred of patients, it is still much smaller than other multicenter audits (11, 38); as such, our results should be considered as preliminary at this stage. While we have split our data into training and validation set, others may not consider this to be sufficient method for validation of the model. As such we seek validation in databases such as VISTA-Acute in future studies.

## Conclusion

Stroke severity rather than dysphagia or dysphagia screen plays critical role in determining the risk of post stroke pneumonia in this cohort. We cannot exclude the possibility that the use of dysphagia screen had minimized the impact of dysphagia on development of pneumonia in this cohort.

## Author Contributions

TP: design and data analysis; TK, CM, SS, BC, and JL: data collection; TP, HM, AT, and VS: manuscript.

### Conflict of Interest Statement

The authors declare that the research was conducted in the absence of any commercial or financial relationships that could be construed as a potential conflict of interest.

## References

[1] Westendorp WF Nederkoorn PJ Vermeij JD Dijkgraaf MG van de Beek D. Post-stroke infection: a systematic review and meta-analysis. BMC Neurol. (2011) 11:110. 10.1186/1471-2377-11-110 21933425 PMC3185266

[2] Kalra L Irshad S Hodsoll J Simpson M Gulliford M Smithard D. Prophylactic antibiotics after acute stroke for reducing pneumonia in patients with dysphagia (STROKE-INF): a prospective, cluster-randomised, open-label, masked endpoint, controlled clinical trial. Lancet (2015) 386:1835–44. 10.1016/S0140-6736(15)00126-9 26343840

[3] Langdon PC Lee AH Binns CW. High incidence of respiratory infections in 'nil by mouth' tube-fed acute ischemic stroke patients. Neuroepidemiology (2009) 32:107–13. 10.1159/000177036 19039243

[4] Dziewas R Ritter M Schilling M Konrad C Oelenberg S Nabavi DG. Pneumonia in acute stroke patients fed by nasogastric tube. J Neurol Neurosurg Psychiatry (2004) 75:852–6. 10.1136/jnnp.2003.019075 15145999 PMC1739077

[5] Marik PE. Aspiration pneumonitis and aspiration pneumonia. N Engl J Med. (2001) 344:665–71. 10.1056/NEJM200103013440908 11228282

[6] Finucane TE Bynum JP. Use of tube feeding to prevent aspiration pneumonia. Lancet (1996) 348:1421–4. 10.1016/S0140-6736(96)03369-7 8937283

[7] Ekberg O Feinberg MJ. Altered swallowing function in elderly patients without dysphagia: radiologic findings in 56 cases. AJR Am J Roentgenol. (1991) 156:1181–4. 10.2214/ajr.156.6.2028863 2028863

[8] Middleton S McElduff P Ward J Grimshaw JM Dale S D'Este C. Implementation of evidence-based treatment protocols to manage fever, hyperglycaemia, and swallowing dysfunction in acute stroke (QASC): a cluster randomised controlled trial. Lancet (2011) 378:1699–706. 10.1016/S0140-6736(11)61485-2 21996470

[9] Hong KS Bang OY Kim JS Heo JH Yu KH Bae HJ. Stroke statistics in korea: part II stroke awareness and acute stroke care, a report from the Korean Stroke Society and Clinical Research Center for Stroke. J Stroke (2013) 15:67–77. 10.5853/jos.2013.15.2.67 24324942 PMC3779666

[10] Donovan NJ Daniels SK Edmiaston J Weinhardt J Summers D Mitchell PH. Dysphagia screening: state of the art: invitational conference proceeding from the State-of-the-Art Nursing Symposium, International Stroke Conference 2012. Stroke (2013) 44:e24–31. 10.1161/STR.0b013e3182877f57 23412377

[11] Bray BD Smith CJ Cloud GC Enderby P James M Paley L. The association between delays in screening for and assessing dysphagia after acute stroke, and the risk of stroke-associated pneumonia. J Neurol Neurosurg Psychiatry (2017) 88:25–30. 10.1136/jnnp-2016-313356 27298147

[12] Powers WJ Rabinstein AA Ackerson T Adeoye OM Bambakidis NC Becker K. 2018 guidelines for the early management of patients with acute ischemic stroke: a guideline for healthcare professionals from the American Heart Association/American Stroke Association. Stroke (2018) 49:e46–110. 10.1161/STR.0000000000000158 29367334

[13] Clinical Guidelines for Stroke Management (2017). Available online at: https://informme.org.au/Guidelines/Clinical-Guidelines-for-Stroke-Management-2017 (Accessed June 27, 2018).

[14] Luker JA Wall K Bernhardt J Edwards I Grimmer-Somers K. Measuring the quality of dysphagia management practices following stroke: a systematic review. Int J Stroke (2010) 5:466–76. 10.1111/j.1747-4949.2010.00488.x 21050403

[15] Smith EE Kent DM Bulsara KR Leung LY Lichtman JH Reeves MJ. Effect of dysphagia screening strategies on clinical outcomes after stroke: a systematic review for the 2018 guidelines for the early management of patients with acute ischemic stroke. Stroke (2018) 49:e123–8. 10.1161/STR.0000000000000159 29367332

[16] Geeganage C Beavan J Ellender S Bath PM. Interventions for dysphagia and nutritional support in acute and subacute stroke. Cochrane Database Syst Rev. (2012) 10:CD000323. 10.1002/14651858.CD000323.pub2 23076886

[17] Charlson ME Sax FL MacKenzie CR Braham RL Fields SD Douglas RG Jr. Morbidity during hospitalization: can we predict it? J Chronic Dis. (1987) 40:705–12. 3110198 10.1016/0021-9681(87)90107-x

[18] Victorian Dysphagia Screening Model ASSIST Tool (2018). Available online at: https://www2.health.vic.gov.au/getfile/?sc_itemid=%7b0CCF9F67-ED96-42B5-A6E3-D963244EE9B6%7d&title=Victorian%20Dysphagia%20Screening%20Model%20ASSIST%20tool (Accessed June 27, 2018).

[19] Hankey G Warlow C Evolution of the concepts of TIAs. In: Hankey G, editor. Transient Ischaemic Attacks of the Brain and Eye. London: WB Saunders (1994). p. 1–9.

[20] Coutts SB Modi J Patel SK Aram H Demchuk AM Goyal M. What causes disability after transient ischemic attack and minor stroke?: results from the CT and MRI in the Triage of TIA and minor Cerebrovascular Events to Identify High Risk Patients (CATCH) study. Stroke (2012) 43:3018–22. 10.1161/STROKEAHA.112.665141 22984013

[21] Shapley LS A Value for N-Person games. In: Kuhn HW Tucker AW, editors. Contributions to the Theory of Games II. Princeton, NJ: Princeton University Press (1953). p. 307–17.

[22] Štrumbelj E Kononenko I Explaining prediction models and individual predictions with feature contributions. Knowl Inf Syst. (2014) 41:647–65. 10.1007/s10115-013-0679-x

[23] Lipovetsky S Entropy criterion in logistic regression and shapley value of predictors. J Modern Appl Stat Methods (2006) 5:85–106. 10.22237/jmasm/1146456480

[24] Hothorn T Hornik K Zeileis A Unbiased recursive partitioning: a conditional inference framework. J Comput Graph Stat. (2006) 15:651–74. 10.1198/106186006X133933

[25] Dirnagl U Klehmet J Braun JS Harms H Meisel C Ziemssen T. Stroke-induced immunodepression: experimental evidence and clinical relevance. Stroke (2007) 38:770–73. 10.1161/01.STR.0000251441.89665.bc 17261736

[26] Hug A Dalpke A Wieczorek N Giese T Lorenz A Auffarth G. Infarct volume is a major determiner of post-stroke immune cell function and susceptibility to infection. Stroke (2009) 40:3226–32. 10.1161/STROKEAHA.109.557967 19661470

[27] Hannawi Y Hannawi B Rao CP Suarez JI Bershad EM. Stroke-associated pneumonia: major advances and obstacles. Cerebrovasc Dis. (2013) 35:430–43. 10.1159/000350199 23735757

[28] Stanley D Mason LJ Mackin KE Srikhanta YN Lyras D Prakash MD. Translocation and dissemination of commensal bacteria in post-stroke infection. Nat Med. (2016) 22:1277–84. 10.1038/nm.4194 27694934

[29] Prass K Braun JS Dirnagl U Meisel C Meisel A. Stroke propagates bacterial aspiration to pneumonia in a model of cerebral ischemia. Stroke (2006) 37:2607–12. 10.1161/01.STR.0000240409.68739.2b 16946159

[30] Sykora M Siarnik P Diedler J Collaborators VA. beta-blockers, pneumonia, and outcome after ischemic stroke: evidence from virtual international stroke trials archive. Stroke (2015) 46:1269–74. 10.1161/STROKEAHA.114.008260 25899243

[31] Masrur S Smith EE Saver JL Reeves MJ Bhatt DL Zhao X. Dysphagia screening and hospital-acquired pneumonia in patients with acute ischemic stroke: findings from Get with the Guidelines–Stroke. J Stroke Cerebrovasc Dis. (2013) 22:e301–9. 10.1016/j.jstrokecerebrovasdis.2012.11.013 23305674

[32] Warusevitane A Karunatilake D Sim J Lally F Roffe C. Safety and effect of metoclopramide to prevent pneumonia in patients with stroke fed via nasogastric tubes trial. Stroke (2015) 46:454–60. 10.1161/STROKEAHA.114.006639 25516196

[33] Lakshminarayan K Tsai AW Tong X Vazquez G Peacock JM George MG. Utility of dysphagia screening results in predicting poststroke pneumonia. Stroke (2010) 41:2849–54. 10.1161/STROKEAHA.110.597039 20947835 PMC2994997

[34] Hastie T Tibshirani R. Generalized additive models for medical research. Stat Methods Med Res. (1995) 4:187–96. 10.1177/096228029500400302 8548102

[35] Phan TG Chen J Beare R Ma H Clissold B Van Ly J. Classification of different degrees of disability following intracerebral hemorrhage: a decision tree analysis from VISTA-ICH collaboration. Front Neurol. (2017) 8:64. 10.3389/fneur.2017.00064 28293215 PMC5329022

[36] Breiman L Friedman JH Olshen RA Stone CJ Classication and Regression Trees. Belmont, CA: Wadsworth (1983).

[37] Hosmer DW Lemeshow S Applied Logistic Regression. 2nd Ed. John Wiley & Sons (2000). 10.1002/0471722146

[38] Ribeiro PW Cola PC Gatto AR da Silva RG Luvizutto GJ Braga GP. Relationship between Dysphagia, National Institutes of Health Stroke Scale Score, and Predictors of Pneumonia after Ischemic Stroke. J Stroke Cerebrovasc Dis. (2015) 24:2088–94. 10.1016/j.jstrokecerebrovasdis.2015.05.009 26187787

